# Identification of combinatorial host-specific signatures with a potential to affect host adaptation in influenza A H1N1 and H3N2 subtypes

**DOI:** 10.1186/s12864-016-2919-4

**Published:** 2016-07-29

**Authors:** Zeeshan Khaliq, Mikael Leijon, Sándor Belák, Jan Komorowski

**Affiliations:** 1 Department of Cell and Molecular Biology, Computational Biology and Bioinformatics, Science for Life Laboratory, Uppsala University, SE-751 24 Uppsala, Sweden; 2 Department of Virology, Parasitology and Immunobiology (VIP), National Veterinary Institute (SVA), Uppsala, Sweden; 3 OIE Collaborating Centre for the Biotechnology-based Diagnosis of Infectious Diseases in Veterinary Medicine, Ulls väg 2B and 26, SE-756 89 Uppsala, Sweden; 4 Department of Biomedical Sciences and Veterinary Public Health (BVF), Swedish University of Agricultural Sciences (SLU), Uppsala, Sweden; 5 Institute of Computer Science, Polish Academy of Sciences, 01-248 Warszawa, Poland

**Keywords:** Influenza A virus, Host adaptation, Combinatorial signatures, Host-specific signatures, MCFS, Rosetta, Rough sets

## Abstract

**Background:**

The underlying strategies used by influenza A viruses (IAVs) to adapt to new hosts while crossing the species barrier are complex and yet to be understood completely. Several studies have been published identifying singular genomic signatures that indicate such a host switch. The complexity of the problem suggested that in addition to the singular signatures, there might be a combinatorial use of such genomic features, in nature, defining adaptation to hosts.

**Results:**

We used computational rule-based modeling to identify combinatorial sets of interacting amino acid (aa) residues in 12 proteins of IAVs of H1N1 and H3N2 subtypes. We built highly accurate rule-based models for each protein that could differentiate between viral aa sequences coming from avian and human hosts. We found 68 host-specific combinations of aa residues, potentially associated to host adaptation on HA, M1, M2, NP, NS1, NEP, PA, PA-X, PB1 and PB2 proteins of the H1N1 subtype and 24 on M1, M2, NEP, PB1 and PB2 proteins of the H3N2 subtypes. In addition to these combinations, we found 132 novel singular aa signatures distributed among all proteins, including the newly discovered PA-X protein, of both subtypes. We showed that HA, NA, NP, NS1, NEP, PA-X and PA proteins of the H1N1 subtype carry H1N1-specific and HA, NA, PA-X, PA, PB1-F2 and PB1 of the H3N2 subtype carry H3N2-specific signatures. M1, M2, PB1-F2, PB1 and PB2 of H1N1 subtype, in addition to H1N1 signatures, also carry H3N2 signatures. Similarly M1, M2, NP, NS1, NEP and PB2 of H3N2 subtype were shown to carry both H3N2 and H1N1 host-specific signatures (HSSs).

**Conclusions:**

To sum it up, we computationally constructed simple IF-THEN rule-based models that could distinguish between aa sequences of avian and human IAVs. From the rules we identified HSSs having a potential to affect the adaptation to specific hosts. The identification of combinatorial HSSs suggests that the process of adaptation of IAVs to a new host is more complex than previously suggested. The present study provides a basis for further detailed studies with the aim to elucidate the molecular mechanisms providing the foundation for the adaptation process.

**Electronic supplementary material:**

The online version of this article (doi:10.1186/s12864-016-2919-4) contains supplementary material, which is available to authorized users.

## Background

Influenza A viruses (IAVs) have been known for a long time to cause disease in a wide range of host species, including humans and various animals. The IAVs are zoonotic pathogens that can infect a broad range of animals from birds to pigs and humans. The interspecies transmission requires that IAVs adapt to the new host and the whole process is facilitated by their high mutation rates [1] and their ability to readily reassort [2]. This can result in epidemics and pandemics with severe consequences for both human and animal life. In addition to the yearly epidemics that proves fatal for at least 250,000 humans worldwide [3], in the 20th century alone, there has been at least five major pandemics; the Spanish flu of 1918, Asian influenza of 1957, Hong Kong influenza of 1968, the age restricted milder Russian flu of the 1977 [4, 5] and the Swine flu of 2009. Thus, new flu epidemics and pandemics are a constant threat. Given our poor understanding of the host adaptation process of the virus, which can be a major factor for such epidemics and pandemics, it is very hard to predict the type of the virus that will cause the coming outbreaks.

The IAVs are usually classified into subtypes based on the two surface glycol-proteins, hemagglutinin (HA) and neuraminidase (NA). To date, 18 types of HA (H1-H18) and 11 types of NA (N1-N11) are known [6–8]. Most of these subtypes have wild birds as their natural hosts. However, occasionally the virus can jump and adapt to a new host species. This cross of the species barrier is proved by the pandemic H1N1, H3N2, H2N2 and the most recent H5N1 and H7N9 subtype outbreaks, which are thought to have evolved from avian or porcine sources [6, 9, 10].

The HA protein plays a crucial part in defining the adaptation of the virus to different hosts since it binds to the receptor providing the entry into host cells. The avian strains of the IAVs are known to attach to a receptor with α2,3-sialic acid linkages while the human strains to a receptor with α2,6-sialic acid linkages [11]. However, other proteins such as the polymerase subunits have also previously been shown to play a role in the adaptation of IAVs to different hosts [12, 13].

Computational methods, like artificial neural networks, support vector machines and random forests, have been used previously to predict hosts of IAVs [14–16]. Furthermore, several other studies have previously been carried out predicting genomic signatures specifying different hosts, both computationally and experimentally [17–23]. Amino acid changes taken one at a time, i.e. singular aa changes, in viral protein sequences between different hosts have been reported by these studies as host-specific signatures (HSSs), some of which likely facilitate the host adaptation process. Despite these findings, the process of adaptation of IAVs in different hosts is still not completely understood. Given the complex nature of the problem we suspected that the adaptation process might not only be dependent on univariate signatures. Essentially, in addition to the proven effects of singular aa residues, there might be a combinatorial use of aa residues in nature that affect the adaptation of IAVs to new hosts.

To this end, for both H1N1 and H3N2 subtypes, we analyzed aa sequences of 12 proteins expressed by the viruses. We have restricted our analyses to these two subtypes because data for both human and avian hosts for all the proteins under-study was available. We built high quality rule-based models, based on rough sets [24], for each of the 12 proteins, predicting hosts from protein sequences. The models consisted of simple IF-THEN rules that lend themselves to easy interpretation. The combinations of aa residues used by the rules were identified as host-specific signatures having the potential to affect the host adaptation of IAVs. In additions to such combinatorial signatures, novel singular signatures were also identified from the rules. The singular and, especially, the combinatorial signatures provide novel insights into the complex host adaptation process of the IAVs.

## Results

### Feature selection reduces the number of features needed to discern between hosts

Monte Carlo Feature Selection (MCFS) [25] was used to obtain a ranked list of significant features, here significantly informative aa positions in all the proteins for both subtypes, that best discern between the hosts. This step helped us remove any kind of noise that could have been in the data. More importantly, the use of MCFS considerably reduced the number of aa positions to be analyzed further, as shown in Table 1. The HA protein had 628 positions to start with and after running MCFS on the data, we were left with 115 and 88 positions for H1N1 and H3N2 subtypes, respectively (81.7 and 86 % reduction in the number aa positions). On average there was a 79.8 % reduction in the number of aa positions across all the proteins for H1N1 subtype and 82.8 % for the H3N2 subtype (Table 1). Only the significant features were used for further analysis in this study. The ranked lists of the significant features are provided as a supplementary file (see Additional file 1).

**Table 1:** The training data

	Nr. of sequences for each subtype					Features after MCFS	
	H1N1		H3N2				
Protein	Avian	Human	Avian	Human	Total features	H1N1	H3N2
HA	214	5205	164	3715	628	115	88
NA	205	3093	173	3412	517	93	79
NS1	150	1258	150	1176	249	98	85
NEP	61	407	54	299	124	31	26
NP	125	839	93	773	506	61	69
M1	45	467	42	355	275	18	15
M2	65	461	64	503	98	25	23
PA	192	1677	143	1358	726	65	47
PA-X	57	164	45	244	252	28	24
PB1	171	1654	132	1347	762	59	33
PB2	184	1817	136	1297	776	52	42
PB1-F2	151	224	112	737	101	64	54

Total Features are the total number of aa positions that are investigated. Features after MCFS are the aa positions that are ranked significant, i.e. having power to discriminate avian from human sequences

### Rule-based models for each protein

Since the number of sequences belonging to human and avian hosts were not balanced in the training data of either subtype (Table 1), we balanced the data sets by a method called under-sampling, as described in detail in Methods. For data sets of each protein and each subtype we created 100 under-sampled subsets. Each of these subsets was used to build a classifier, consisting of IF-THEN rules, whose performance was assessed by a 10-fold cross-validation (Table 2). HA classifiers for H1N1 and non-structural protein 1 (NS1) classifiers for H3N2 subtypes were the best ones with a mean accuracy of 98 and 98.9 %, respectively. Nuclear export protein (NEP) classifiers of the H1N1 subtype and matrix protein 1 (M1) classifiers of the H3N2 subtype had lowest mean accuracy of 83.4 and 88.8 %, respectively.

**Table 2:** 10-fold cross-validation accuracies

	Mean accuracy (%)	
Protein	H1N1	H3N2
HA	98	98.7
M1	87.7	88.8
M2	87.6	92.9
NA	93.9	98.6
NP	93	97.3
NS1	93.1	98.9
NEP	83.4	95.3
PA	95.1	97.9
PA-X	95.9	97.7
PB1	94.7	95.1
PB1F2	95.5	92.3
PB2	95.9	97.5

Cross-validation accuracies of the 100 classifiers were averaged

For each protein of each subtype a single rule-based model containing only the most significant rules from their respective 100 classifiers was inferred (Methods). We then reclassified the training data of each protein with its respective rule-based model to get an idea of its performance in terms of classification of human and avian sequences. Polymerase acidic protein X (PA-X), which is a frame-shift product of the third RNA segment, HA and NEP (NS2) models performed the best (Matthews correlation coefficient (MCC) = 1, MCC = 0.993, MCC = 0.988, respectively) among the H3N2 models while HA, NA and NS1 models performed the best among the H1N1 models (MCC = 0.961, MCC = 0.95, MCC = 0.954, respectively) (Table 3). The poorest of the H1N1 models was the PA-X protein model (MCC = 0.856) and of the H3N2 models was the polymerase basic protein F2 (PB1-F2) protein model (MCC = 0.861). The complete HA H1N1 rule-based model is shown in Table 4. Models for the remaining proteins for both subtypes are provided as supplementary material (Additional file 2).

**Table 3:** Performance of the models on their corresponding complete data sets

	H1N1			H3N2		
Protein	Sensitivity	Specificity	MCC	Sensitivity	Specificity	MCC
HA	0.999	0.953	0.961	1	0.987	0.993
M1	1	0.881	0.934	0.994	1	0.971
M2	1	0.859	0.918	0.996	0.873	0.908
NA	1	0.907	0.95	1	0.908	0.95
NP	1	0.864	0.92	0.994	0.957	0.946
NS1	0.998	0.932	0.954	0.991	0.993	0.96
NEP	0.995	0.883	0.912	0.997	1	0.988
PA-X	0.901	1	0.856	1	1	1
PA	0.972	0.979	0.892	0.996	0.979	0.969
PB1-F2	0.91	0.987	0.884	0.999	0.778	0.861
PB1	0.993	0.93	0.923	1	0.879	0.932
PB2	0.989	0.984	0.935	0.996	0.985	0.972

Sensitivity is the ability to correctly predict human sequences and specificity is the ability to correctly predict avian sequences where 1 means perfect prediction and 0 means no correct prediction. Matthews correlation coefficient (MCC) value is a measure of how well the model performs overall where 1 means a perfect classification, 0 is for a prediction no better than random and −1 indicates a total disagreement between predictions and observations. “na” means the measure could not be calculated for the given model

**Table 4:** Example rule-based model

Rule	Accuracy (%)	Support	Decision coverage (%)
IF P435 = I THEN host = Human	99.9	5128	98.4
IF P200 = S THEN host = Human	99.9	4052	77.8
IF P10 = Y THEN host = Human	99.8	3998	76.7
IF P88 = S THEN host = Human	99.9	3989	76.5
IF P6 = V THEN host = Human	99.8	3936	75.5
IF P222 = R THEN host = Human	99.9	3823	73.4
IF P220 = T THEN host = Human	100.0	3584	68.8
IF P516 = K THEN host = Human	99.9	1818	34.9
IF P200 = P and P222 = K THEN host = Avian	91.3	229	97.7
IF P130 = K THEN host = Avian	91.3	218	93.0
IF P2 = E and P222 = K THEN host = Avian	96.2	208	93.5
IF P137 = A and P544 = L THEN host = Avian	96.1	205	92.1
IF P78 = L and P435 = V THEN host = Avian	97.1	204	92.5
IF P9 = F THEN host = Avian	98.5	204	93.9
IF P6 = F THEN host = Avian	98.2	169	77.6
IF P14 = V THEN host = Avian	99.4	165	76.6
IF P173 = T THEN host = Avian	98.7	158	72.9

The model presented here is for the HA protein of the H1N1 subtypeModels for the other proteins of both the subtypes are listed in Additional file 2

To further verify the validity of the rule-based models created, we tested them on new, unseen data. This data was protein sequences published at the NCBI resource between 30th of November 2014 and 16th of April 2015. For the H1N1 subtype, the rule-based models of M1, nucleoprotein (NP), NS1, NEP (also called non-structural protein 2 (NS2)), PB1-F2, polymerase basic protein 1 (PB1) and polymerase basic protein 2 (PB2) provided perfect classification (i.e. all the sequences were correctly classified). For the H3N2 subtype data, the models of HA, M1, NP, NS1, NEP (NS2), polymerase acidic protein (PA), PB1 and PB2 also gave a perfect classification. Table 5 shows the performance of all rule-based models on the unseen data. A list of names of the viruses that could not be classified or were miss-classified for both subtypes is given in Additional file 3.

**Table 5:** Performance of the rule-based models on the new, unseen data

	Human sequences		Avian sequences		
Protein	Total	Correctly classified	Total	Correctly classified	Accuracy (%)
HA-H1N1	108	105	2	2	97.3
HA-H3N2	73	73	4	4	100.0
M1-H1N1	25	25	0	0	100.0
M1-H3N2	8	7	0	0	87.5
M2-H1N1	30	26	2	2	87.5
M2-H3N2	22	16	3	3	76.0
NA-H1N1	33	33	2	1	97.1
NA-H3N2	46	46	4	3	98.0
NP-H1N1	13	13	2	2	100.0
NP-H3N2	8	8	4	4	100.0
NS1-H1N1	31	31	2	2	100.0
NS1-H3N2	19	19	3	3	100.0
NEP-H1N1	12	12	2	2	100.0
NEP-H3N2	8	8	2	2	100.0
PAX-H1N1	18	14	2	2	80.0
PAX-H3N2	7	7	0	0	100.0
PA-H1N1	34	29	2	2	86.1
PA-H3N2	23	23	4	4	100.0
PB1F2-H1N1	3	3	2	2	100.0
PB1F2-H3N2	9	8	4	0	61.5
PB1-H1N1	27	27	1	1	100.0
PB1-H3N2	20	20	1	1	100.0
PB2-H1N1	29	29	2	2	100.0
PB2-H3N2	16	16	3	3	100.0

### Predicted host-specific signatures

The rule-based models allowed us to further interpret them and see how they differentiated viral avian from viral human sequences. Each of the models was analyzed separately for HSSs. The constituent rules of a model associated aa residues at specific positions with an avian or human host. The confidence in these associations is shown as the accuracy, support and the decision coverage shown in the rule-based models. For the combinations in our models we also calculated a combinatorial accuracy gain (CAG), which is the percentage points gain in accuracy of the combination as compared to the average of the accuracies of its constituent singular conditions when taken independently.

#### Combinatorial signatures

**Fig. 1:**
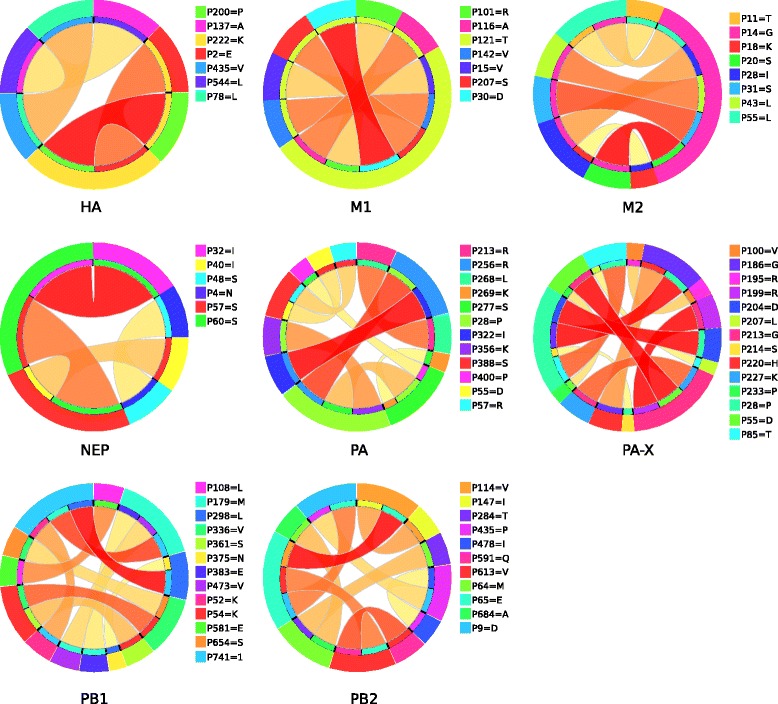
Ciruvis diagrams of combinations from the rules of H1N1 models. Models having at least three combinations are shown. The outer circle shows the positions. The inner circle shows the position or positions to which the position of the outer circle is connected. The edges show these connections. The width and color of the edges are related to the connection score (*low = yellow and thin, high = red and thick*). The width of an outer position is the sum of all connections to it, scaled so that all positions together cover the whole circle [26]

**Fig. 2:**
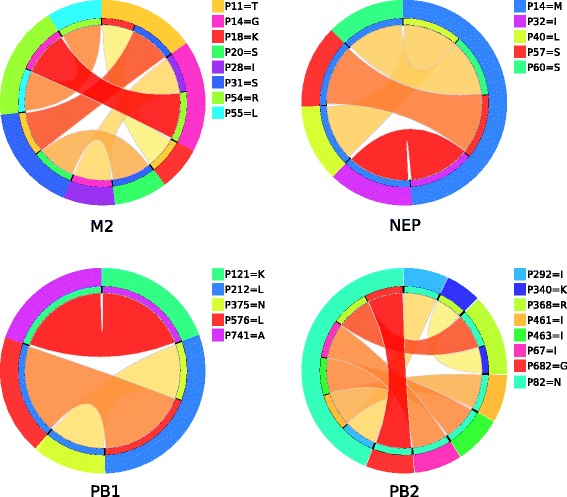
Ciruvis diagrams of combinations from the rules of H3N2 models. Models having at least three combinations are shown. The outer circle shows the positions. The inner circle shows the position or positions to which the position of the outer circle is connected. The edges show these connections. The width and color of the edges are related to the connection score (*low = yellow and thin, high = red and thick*). The width of an outer position is the sum of all connections to it, scaled so that all positions together cover the whole circle [26]

As expected we found aa combinations, i.e. the combinatorial HSSs, in HA, M1, matrix protein 2 (M2), NP, NS1, NEP (NS2), PA, PA-X, PB1 and PB2 proteins to be associated with specific hosts in the H1N1 subtype. In the H3N2 subtype, we found combinations in M1, M2, NEP, PB1 and PB2 proteins. A complete set of the combinatorial HSSs for both subtypes is given in a supplementary file (see Additional file 4: Combinations_from_rules). Ciruvis diagrams [26] for visualization of combinations of interacting amino acids were used to illustrate the cases of three or more combinations in the models of both subtypes associated with the avian hosts (see Figs. 1 and 2).

Residues 14G of the M2 H1N1 model and 82 N of the PB2 H3N2 model were the most connected ones interacting with six other aa residues each. Amino acid residues having interactions with more than one other residue, in both the subtypes are listed in Table 6. These strongly interacting residues might be relatively more essential to host adaptation than the less connected ones.

**Table 6:** Amino acid residues having the most interactions in the models of both subtypes

Subtype	Protein	Positions	Number of interactions
H1N1	HA	222K	2
	M1	121T	5
	M2	14G	6
	NEP	57S, 60S	2
	PA	28P, 277S	3
	PA-X	28P	4
	PB1	179M, 741A	3
	PB2	65E	3
H3N2	M1	101R	2
	M2	11T, 14G, 31S, 54R	2
	NEP	14M	4
	PB1	212L	2
	PB2	82N	6

#### Singular (linear) signatures

Previous studies [17–23] mostly found the adaptation signatures on the internal proteins and did not look into surface glycoproteins (HA and NA). In contrast, we found singular HSSs on all the proteins of both subtypes, including the HA, NA and the newly discovered PA-X proteins. In total, 189 singular HSSs were found, in both subtypes combined. Out of these, 132 signatures were novel and not reported by the previous studies (Table 7). A complete list of singular signatures is given in the supplementary material (see Additional file 4: singletons_H3N2, singletons_H1N1).

**Table 7:** Novel singular aa positions associated to host adaptation

Protein	Novel singular positions
HA	6,9,10,14,23,47,66,69,78,88,91,94,130,173,189,200,220,222,435,516
M1	30,116,142,207,209
M2	13,16,31,36,43,51,54
NA	16,18,19,23,30,40,42,44,46,47,74,79,147,150,157,166,232,285,341,344,351,369,372,389,397,435,437,466
NP	31,53,98,146,444,450,498
NS1	6,7,14,23,27,28,74,123,152,192,220,226
NS2	6,7,14,32,34,48,83,86
PA	85,323,336,348,362,300
PAX	28,85,210,233
PB1	12,54,59,113,175,212,339,435,576,586,587,619,709
PB1F2	3,6,12,17,21,25,26,27,28,33,47,52,54,57,58,60,62,65,82
PB2	54,65,354

#### Specific aa changes predicted to be associated with host adaptation

Some of the rules from our models associated different residues at the same aa positions with avian and human hosts. This can be seen as a mutation (aa change) potentially affecting the adaptation of the viral proteins to a specific host. Eight mutations were found for the H1N1 subtype and 10 for the H3N2 one. In the H1N1 subtype, mutations F6V in HA, P46T and L74V in NA, I6M in both NS1 and NEP and L58- in PB1-F2 were novel. In the H3N2 subtype, mutations R78E in HA, A30I, N40Y and I44S in NA, P28L and R57Q in PA and P28L in PA-X were not identified in the previous studies. Table 8 shows all such mutations in both subtypes.

**Table 8:** Amino acid changes associated with host adaptation

H1N1				H3N2			
Protein	Position	Avian	Human	Protein	Position	Avian	Human
HA	6	F	V	HA	78	R	E
NA	46	P	T	NA	30	A	I
	74	L	V		40	N	Y
NP	100	R	I,V		44	I	S
NS1	6	I	M	NP	16	G	D
NEP	6	I	M	PA-X	28	P	L
PB1-F2	58	L	-	PA	28	P	L
PB2	588	A	I		57	R	Q
				PB2	9	D	N
					64	M	T

### HSSs are not specific to phylogenetic sub-clades within a host

The support and the decision coverage of the rules showed whether the HSSs identified were specific to sub-clades or were more general i.e. spread out across the sub-clades. The higher decision coverage indicated more generality of the rule. For example, the top five rules for the avian class have the following very high decision coverage: rule1 – 98.5 %, rule2 – 98.5 %, rule3 – 97.8 %, rule4 – 98.5 % and rule5 – 97.8 %. It follows that the rules are general. To further illustrate this generality, and to show the diversity in our training data set, a phylogenetic analysis was carried out (Additional file 5). Top five rules specifying each host were mapped onto the created phylogenetic trees, separately for each host, for all the proteins of both subtypes.

**Fig. 3:**
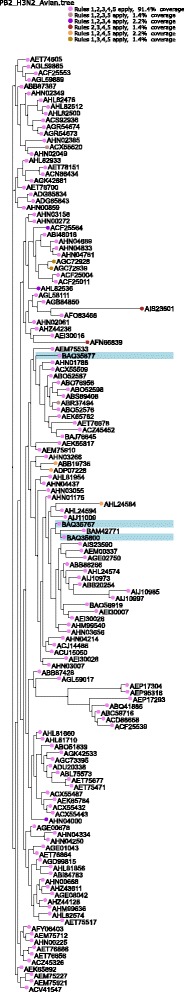
Phylogeny of PB2 H3N2 protein of avian hosts annotated with top 5 avian rules form the PB2 H3N2 model. Each sequences is represented by its GeneBank accession. The violet nodes mark the sequences that supports rule 1,2,3,4 and 5, which are 91.4 % of the total sequences. Similarly the DarkViolet nodes mark the sequences that support rule 1, 2, 3 and 4 but lacks support for rule 5, which are 2.2 % of the total sequences. The nodes with a LightBlue background are the new, unseen sequences. The unmarked nodes do not support the top 5 rules, and were either supporting rules other than the top 5 or were not classified by the models

As an example, consider the avian PB2 H3N2 tree (Fig. 3). 91.4 % of the sequences are covered by rule 1, 2, 3, 4 and 5, which is illustrated by the violet coloring of the leaves in the tree. Only, 1.4 % of the sequences were not covered by rule4, yet they were covered by rule 1, 2, 3, and 5, and similarly for the remaining coverage. For the corresponding human tree, the figures are 89.3 % coverage for the top five human rules. One can see that this generality prevails in all other proteins.

### Validity of HSSs across H1N1 and H3N2 subtypes

To see whether the HSSs identified in the H1N1 subtype could predict hosts of the H3N2 subtype aa sequences and vice versa, we classified H3N2 subtype data with H1N1 models and H1N1 subtype data with H3N2 models. Good classifications meant that the rules (and consequently the HSSs) generated for one subtype were valid for the other one. Bad classifications meant that the rules of one subtype did not hold for the data of the other subtype and hence no cross-subtype signatures validity. Both HA and NA H1N1 models were bad classifiers for the HA and NA of the H3N2 type data, respectively since they failed to distinguish avian sequences in the data in both cases (Sp = 0) (Table 9). It should be kept in mind that the outcome *human* was considered positive outcome and the outcome *avian* considered as a negative one. The PA-X H1N1 model could not recognize human sequences in the PA-X H3N2 data (Sn = 0). Furthermore, the models of PA, PB1-F2 and PB1 proteins of H1N1 subtype were bad classifiers of the H3N2 data (MCC = −0.11, MCC = 0.056, MCC = 0.302), specifically failing to identify sequences coming from human hosts (Sn = 0.021, Sn = 0.023, Sn = 0.563). This meant that H1N1 HSSs in the models of HA, NA, PA-X, PA, PB1-F2 and PB1 proteins were not valid for H3N2 subtype data and these proteins of the H3N2 subtype carried only H3N2-specific HSSs. Contrary to this, the H1N1 models of M1, M2, NP, NS1, NEP and PB2 proteins were able to distinguish between H3N2 subtype sequences coming from avian and human sources reasonably well (Sn = 0.97–1.0; Sp = 0.64–0.94; MCC = 0.776–0.941). It proved that these proteins of the H3N2 subtype, in addition to the stronger H3N2 HSSs, also carried H1N1 HSSs.

**Table 9:** Performance of the H1N1 models on H3N2 data and vice versa

	Protein	Sensitivity	Specificity	MCC
H3N2 data - H1N1 models	HA	1	0	na
	M1	1	0.895	0.941
	M2	1	0.73	0.84
	NA	1	0	na
	NP	1	0.882	0.932
	NS1	1	0.747	0.85
	NEP	1	0.648	0.78
	PA-X	0	1	na
	PA	0.021	0.93	−0.11
	PB1-F2	0.023	1	0.056
	PB1	0.563	0.909	0.302
	PB2	0.979	0.949	0.873
H1N1 data - H3N2 models	HA	0	na	na
	M1	0.957	0.975	0.885
	M2	0.987	0.766	0.804
	NA	1	0	−0.004
	NP	0.364	0.984	0.251
	NS1	0.365	0.993	0.237
	NEP	0.027	1	0.061
	PA-X	0.201	0.982	0.223
	PA	0.247	0.995	0.177
	PB1-F2	0.991	0.804	0.832
	PB1	0.992	0.877	0.888
	PB2	0.956	0.951	0.786

Sensitivity is the ability to correctly predict human sequences and specificity is the ability to correctly predict avian sequences where 1 means perfect prediction and 0 means no correct prediction. Matthews correlation coefficient (MCC) value is a measure of how well the model performs overall where 1 means a perfect classification, 0 is for a prediction no better than random and −1 indicates a total disagreement between predictions and observations. “na” means the measure could not be calculated for the given model

The H3N2 models of HA, NA, NP, NS1, NEP, PA-X and PA proteins could not classify avian and human sequences of H1N1 subtype correctly (MCC = −0.004–0.251). This means that these proteins of the H1N1 subtype carried H1N1-specific HSSs. Whereas the successful classifications of H1N1 subtype data of M1, M2, PB1-F2, PB1 and PB2 proteins by the respective H3N2 models (MCC = 0.788–0.888; Sn = 0.956–0.992; Sp = 0.766–0.951) proved that these H1N1 proteins carried both H1N1 and H3N2 signatures.

**Fig. 4:**
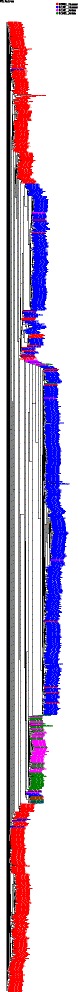
Phylogenetic tree of the M1 protein from sequences of both subtypes and both hosts. Both the subtypes and the hosts are combined into a single tree. It can be seen that human sequences of both the subtypes form their own distinct clades. The avian sequences, on the other hand, fell into a single clade

For predicting hosts from an aa sequence we analyzed positions specified by the rules with the remainder of the sequence not taken into account. This meant the existence of a sequence in one or the other phylogenetic clade would not affect the validation of the predicted signatures across subtypes. To prove this point we included sequences of both subtypes into one single phylogeny for all the proteins. In the M1 phylogeny (Fig. 4) the human sequences from both the subtypes formed distinct clades. The avian sequences, on the other hand, did not form separate clades but formed a single clade. This meant that the human sequences were relatively more different between the subtypes than the avian sequences. Across subtype validation of HSSs of the M1 protein proved that the H1N1 signatures were valid in H3N2 data, meaning that we could predict the hosts of H3N2 human sequences using H1N1 signatures. The reason is that the sequences were similar in the analyzed positions. The remainders of the sequence, with comparatively low sequence similarity, did not affect the prediction process. On the other hand, we could also predict the hosts of the H3N2 avian sequences by using H1N1 signatures where the remainders of the sequences had more sequence similarity. And conversely, the H3N2 signatures were also valid for H1N1.

Furthermore, the clades in the NP phylogeny were more or less similar to the M1 phylogeny (Additional file 6). H1N1 signatures were valid for H3N2 sequences but the converse did not hold, i.e. the H3N2 signatures were not valid for the H1N1 sequences.

For the HA and NA phylogeny (Additional file 6), the different subtypes and hosts formed separate clades. The cross-subtype validation of signatures failed for these two proteins. However, this failure was not due to the underlying phylogeny; rather the signatures of one subtype could not predict hosts in the other subtype.

It follows that to predict hosts, our method indeed analyzes specific positions in the sequences as specified by the rules, and the remainders of the sequences or the underlying phylogenies do not affect the predictions.

## Discussion

Our models performed reasonably well since all of them had an average accuracy of more than 90 % in the 10-fold cross validation except NEP (NS2), M1 and M2 protein models of the H1N1 type (Accuracy: 83.4, 87.7 and 87.6 %, respectively) and M1 protein model of the H3N2 type (Accuracy 88.8 %) (Table 2). The reason for the somewhat lower accuracies of the above exceptions could be due to either the lack of training sequences from which the models learn or to the absence of stronger HSSs in these sequences.

In previous studies [17–23], signatures of adaptation were mostly found on the internal proteins, especially in viral ribonucleoprotein complexes consisting of viral polymerases and NP. The fact that we were able to build high quality models for all the proteins for both subtypes, indicated that all the proteins, including the highly variable HA and NA proteins and the recently discovered PA-X protein, carry HSSs. A major difference between our models and the ones previously reported [14–16] is that the previous models were black box classifiers whereas our models are transparent. Black box classifiers give classification but do not provide any straightforward possibility to identify which parameters and for which values a classification is obtained. Transparent classifiers allow explicit analysis of the model, i.e. the features and their values, for each classified object. The models created in this study used aa positions as features and aa residues at those positions as the values for those features, hence lending themselves for easy interpretation and further analysis.

In comparison to previous studies, we identified a larger number of singular HSSs. One reason is that our method requires aa residue at a particular position to be more or less conserved/persistent in one host only. The same position may either have another persistent residue at this position or not have a persistent residue at all. For example, if at a given position in human hosts there is a conserved “Leucine”, our method selects this position as a signature of human hosts. The previous studies required that this position be fully or partially conserved in the avian hosts, too, which leads them to having a smaller number of signatures. Furthermore, the previous studies did not analyze the subtypes and the proteins separately. Not limiting analyses to particular subtypes leads to identifying more generic signatures but may loose signatures that are stronger in a subtype-specific manner. Analyzing all the proteins at the same time also results into a smaller number of signatures since the stronger signatures from some proteins may shadow the weaker signatures from the other proteins. We also had more data in some cases. Taubenberger et al., [17] had 105, 91 and 83 sequences for proteins PA, PB1 and PB2 while we had 1869,1825 and 2001 in H1N1 and 1501,1479 and 1436 sequences in H3N2 subtype for those proteins respectively. Finkelstein et al., [20] had ~9 times less data for HA, ~6 times less for NA and ~4 times less data for each of the polymerases. Allen et al. [21] had only 281 human sequences and 560 avian sequences for all the proteins.

In addition to singular HSSs, we also identified combinatorial HSSs. Indeed, it is the very first time that combinatorial HSSs are reported in this context. These HSSs are shown as conjunctive rules, i.e., rules with more than one condition in the IF part. It appeared that some aa residues were part of more than one combination in our models. This may suggest that these residues are potentially more important in establishing host adaptation then the ones appearing in one combination only (Table 6).

In the M2 H1N1 model, the combinations associated with avian hosts had a Glycine (G) residue at position 14 while the combinations for human hosts had a Glutamic acid (E) in the same position. Similarly, in PB2 H3N2 model, Arginine (R) at position 340 was associated to avian hosts while Lysine (K) residue at the same position to human hosts. It seems that the mutations G14E in M2 H1N1 and R340K in PB2 H3N2 model potentially facilitate the shift of hosts from avian to human. However, these residues always appear in combination with other residues and therefore they are HSSs only in combinations and not individually. The reason is obvious. The confidence measures (accuracy, support and decision-coverage) were calculated for the combination as a whole. We do not report such mutations in our list of mutations, although they indicate an effect. The functions of these combinations at a molecular level are not understood yet, but they provide a novel and interesting perspective of looking at sequence-based host adaptation.

The method used in this study is also a sequence-based method like phylogeny. Phylogeny puts sequences in clades and sub-clades based on the similarity or difference of the complete sequences but it does not output how exactly or at what positions the sequences are different. Our classification method identifies the exact aa differences (the HSSs) between the sets of sequences. For the sake of an example, let us assume that at a given position the avian viruses carry a conserved Methionine and at the same position in the human viruses there is a conserved Alanine. This position will be identified by our method as a host-specific signature. The remainder of the sequence does not affect the identification process. In order to simplify the argument we consider two extreme cases. a) The remainders of the sequences are identical. The two sequences will most likely be put into the same phylogenetic clade. However, our method will select the afore-mentioned position because it differentiates avian and human viruses. b) The remainders of the sequences differ entirely. The sequences will be assigned to different phylogenetic clades, while our method will select the said position since it nonetheless differentiates the viruses. It follows that our method is invariant of the underlying phylogeny. In practice, however, since we are predicting the variable host that is not independent of phylogeny, some of the HSSs discussed may inform on the phylogeny.

HA and NA of both subtypes were found to be only carrying subtype-specific HSSs. This goes well with the current knowledge that these two proteins are the most diverse proteins that are specifically adapted to interact with the host cell. M1, M2 and PB2 are shown to be the most conserved proteins from the point of view of host specifying genomic signatures since they carried the HSSs valid for both subtypes.

The HSSs found in this study were also considered in other contexts in other studies such as viral viability and antiviral resistances. For instance, positions 30, 142, 207 and 209 occurring in the H1N1 M1 models have been previously shown to affect viral production when mutated [27], while mutation S31N derived from M2 models is a known marker of amantadine resistance [28–31]. Table 10 lists all the aa residues and their descriptions as found in different contexts in the literature. All these different contexts, that the aa residues from our models are described in, show that they affect the fitness of the viruses in one or the other way, which in turn facilitates their adaptation to the new environment or hosts.

**Table 10:** Amino acid positions discussed in literature from the models of both the subtypes for all proteins

Protein	Positions	Description
M1	115,121,137	Known signatures of host-adaptation [19, 22, 23]
	30,142,207,209	Affecting viral production on mutation [27]
	121	Affecting viral replication [45]
	101	Determinant of temperature sensitivity [46], located in a transcription inhibition site [47] and is also interacting with NEP [48]
M2	11,14,18,20,28,55,57,78,82,89,93	Known signatures of host-adaptation [19, 22, 23, 49]
	31	S31N is a known marker for amantadine resistance [28–31]
	18,20	Lie next to 17,19 which forms a di-sulphide bond [50]
NS1	18,21,22,53,60,70,81,112,114,171,215,227	Known signatures of host-adaptation [18, 20–23, 51]
	215	Required for Crk/CrL-SH3 binding [52]
	123	Necessary for interaction with PKR, resulting in an inhibition of eIF2alpha phosphorylation [53]
	95	Along with others, has been shown to be necessary for binding p85beta and activating PI3K signaling [54, 55]
	220	Part of nuclear localization signal 2 essential for the importin-alpha binding [56]
NEP(NS2)	57,60,70,107	Known signatures of host-adaptation [18, 19, 22, 23, 57]
NP	16,33,100,214,283,313,351,353,357,422	Known signatures of host-adaptation [19–23, 58]
	16	D16G shown to decrease pathogenicity several fold [59]
PA	28,55,57,65,256,268,277,356,382,400,409	Known signatures of host-adaptation [19–23, 58]
	85,336	Residues 85I and 336 M are deemed important for enhanced polymerase activity in mammalian cells [60]
	57,65,85	Shown to be involved in suppressing the host cell protein synthesis during infection [61]
PB1	52,179,216,298,327,336,361,375,581,741	Known signatures of host-adaptation [17, 19, 22, 23, 58]
	581	Shown to be conferring temperature sensitivity to human influenza virus vaccine strains [62]
	473	Mutation at position 473 has been shown to decrease polymerase activity [63]
PB2	9,44,64,81,105,271,292,368,453,588,613,682,684	Known signatures of host-adaptation [19, 20, 22, 23, 58]
	591	591Q is known to mimic the effect of 627 K [64, 65]
	271	271A shown to increase polymerase activity in mammalian cells [66]
	271,588	Also been shown to be host range determinants [67]
PB1-F2	16,23,42,66,70,73,76	Known signatures of host-adaptation [18, 23]
	66	Linked with affecting pathogenicity [68]
NA	46,47,74,147,157,341,351	Under selection pressure with a shift of hosts from birds to humans [58]
	344	Calcium ion binds here that stabilizes the molecule (UniProt: Q9IGQ6).
HA	2,6,9,10,14	Signal peptide domain
	88,173,220,22	Position 71, 159, 206 and 208 of the fully-mature HA with H3-numbering [69]) are part of the antigenic sites Cb, Sb and Ca of the HA protein, respectively [70, 71]

## Conclusions

The highly predictive rule-based models built for 12 proteins for H1N1 and H3N2 subtypes suggest that there are HSSs on all the protein including the diverse HA, NA and the newly discovered PA-X protein that were not previously studied in this context. In addition, the transparent nature of our method allowed us to further investigate our models for how the predictions were actually done. This resulted in lists of predicted singular and combinatorial HSSs. Some of the HSSs identified in this study were already known while others are novel. The ability of our methods to capture combinatorial HSSs that may affect the host adaptation process makes this study unique. We discovered that the surface proteins HA and NA carry subtype-specific HSSs in both subtypes while NP, NS1, NEP, PA-X and PA of the H1N1 subtype and PA-X, PA, PB1-F2 and PB1 of the H3N2 subtype carry subtype-specific HSSs. We showed that M1, M2, PB1-F2, PB1 and PB2 of the H1N1 subtype carried H1N1 and some additional H3N2 HSSs, and vice versa, M1, M2, NP, NS1, NEP and PB2 of the H3N2 subtype carried H3N2 and some additional H1N1 signatures. The computational results presented here will eventually require further analysis by testing the host-pathogen interactions under laboratory conditions. We believe that the computational analyses provide important support in the characterization of host-pathogen interactions and the proper combination of *in silico* and *in vitro* (probably even *in vivo*) studies will yield important novel information concerning the infection biology of various viruses and other infectious agents.

## Methods

The combined feature selection – rule-based modeling methodology used in this is similar to our previous work where we identified a complete map of potential pathogenicity markers in the H5N1 subtype of the avian influenza A viruses [32].

### Data

The data used to make the models was downloaded from the NCBI flu database found at http://www.ncbi.nlm.nih.gov/genomes/FLU/Database/nph-select.cgi?go=database [33]. Full-length plus (nearly complete, may only miss the start and stop codons) protein sequences of the twelve proteins namely, HA, NA, NP, M1, M2, NS1, NEP (NS2), PA, PA-X, PB1, PB2 and PB1-F2, were separately downloaded as published up till November 30, 2014. Identical sequences were represented by the oldest sequence in the database. For each protein, sequences of the H3N2 and H1N1 subtypes of avian and human hosts were downloaded. Sequences of the mixed subtypes were not included in this study. Table 1 shows the number of sequences for each of the proteins for each subtype. For each protein we combined the sequences of the two subtypes used in this study into a single file and aligned them with MUSCLE (v3.8.31) [34].

### Decision tables

A decision table was created for each of the proteins for both the subtypes. A decision table can be seen as a tabularized form of the aligned FASTA sequences with an extra decision/label column, which in our case was the host information. The first column of the decision tables contained the identifier of the sequence, and the last column was the label/outcome column, the host information in our case and the rest of the columns represented the sequence information corresponding to the aligned FASTA files. The alignment gaps were represented by a ‘?’ in the decision tables. The rows of a decision table were called objects each representing a particular aa sequence and a label. Columns other than the first and the last one were the features.

### Feature selection

MCFS, as described in [25], was used to rank the features of the decision tables with respect to their ability to discern between avian and human hosts. MCFS is implemented as a software package dmLab [35]. MCFS uses a large number of decision trees and assigns a normalized relative importance (RI-norm) score to each feature such that the features contributing more to the discernibility of the outcome gets a higher score. Statistical significance of the RI-norm scores was assessed with a permutation test and significant features (*p* < 0.05), after Bonferroni correction [36], were kept as described in [37]. Only these features were used in the further rule-based model generation.

### Under-sampling the data sets

In the training data for both subtypes, the number of sequences from human hosts was considerably higher than that from the avian hosts. It has previously been shown that this imbalance affects the learning in favor of the dominating class [38]. However to address this problem one can artificially balance the classes [39]. To this end, a technique called under-sampling was used where the sequences belonging to the dominating class were randomly sampled equal to the class having the lesser number of sequences and repeated this step 100 times. In this way for each protein and for each subtype we created 100 subsets where the number of sequences belonging to human and avian hosts were equal. A single rule-based classifier was inferred from each of the subsets, which resulted in 200 classifiers per protein (100 for each subtype). We illustrate the process with the following example.

The data set of the NA protein of the H1N1 subtype had 3093 human and 205 avian sequences, which was a significant imbalance in the number of sequences. From the human set we created subsets by randomly extracting 100 times 205 human sequences and joining them with the 205 avian sequences to create 100 subsets.

### Rough sets and rule-based model generation

Rough set theory [24] was used to produce minimal sets of features that can discern between the objects belonging to different decision classes. ROSETTA [40], a publicly available software system that implements rough sets theory, was used to transform the minimal sets of features into rule-based models [41] that consisted of simple IF-THEN rules. A complete description of rough sets can be found in [42] and the combined MCFS-ROSETTA approach to model generation in bioinformatics is described in [43].

The input data to ROSETTA were the balanced decision tables created in the previous step with only the significant features obtained from applying MCFS. ROSETTA computed approximately minimal subsets of feature combinations that discerned between avian and human hosts with the Johnsons algorithm implemented in ROSETTA. The classifiers were collections of IF-THEN rules. A sample rule from the HA-H1N1 model:

**Table:** 

Rule	Accuracy (%)	Support	Decision coverage (%)
IF P200 = P AND P222 = K THEN host = Avian	91.3	229	97.7

reads as: “**IF***at position 200 there is a Proline residue***AND***at position 222 there is a Lysine residue***THEN***the sequence is from an avian host*”*.*

There is additional information about the rules available. *Support* is the set of sequences (229 sequences) that satisfy the conditions of the left hand side (LHS), i.e. the set of sequences that have a proline residue at position 200 and a lysine residue at position 222. For this rule, *Accuracy* is 91.3 % that is the proportion of correctly classified sequences to the total number of supporting sequences (209/229). Human sequences are considered positive and avian as negatives in this study. The decision coverage for this rule is 97.7 %, which means it correctly classifies 97.7 % of the total avian sequences used to train the classifier. It is calculated as follows:

$$ Decision\  Coverage\ \left(\%\right) = \left(\frac{Accuracy \times Support}{Total\  training\  objects\  of\  the\  decision\  class}\right) \times 100 $$

*Accuracy* × *Support* gives us the total number of sequences that are correctly classified by the rule. Since the rule is for the avian decision class, the total number of avian sequences used to train the classifier was 214. So for the stated rule the decision coverage will be ((0.913*229)/214)*100, which is equal to 97.7 %. The above rule is a conjunctive rule since there is a conjunction of conditions (P200 = P AND P222 = K) in the left hand side (LHS) of the rule. A conjunctive rule captures the combinatorial HSSs. Each conjunctive rule must always be used as combination only, because the support, accuracy and the decision coverage measures are calculated for the conjunction and not for the individual conjuncts. A rule can also be a singleton rule where LHS consists of only a single condition.

The confidence in these classifiers come from the 10-fold cross validation performed in ROSETTA. In a 10-fold cross validation step the input data set is randomly divided into ten equal subsets, say {P1, …, P10}. A classifier is trained on the first nine subsets {P1, …, P9} and then tested on the remaining, P10 subset. In the next run, another classifier is trained on {P1, …, P8, P10} and its performance is tested on the remaining subset, this time P9. Notice that each time the test set is a different one. The process is repeated 10 times and by then each subset has been used once as a test set. The performance of all the classifiers is averaged and presented as a cross-validation accuracy. Such a validation is quite common in machine learning since one becomes more or less assured that the performance of the classifier was not simply by chance.

### Extraction of a single rule-based model for each protein

Rules from all the 100 classifiers were combined into a single file. Duplicates were removed. Among partially identical rules, the one with the highest decision coverage was kept. If the difference of decision coverage was lower than 1 % then the shortest (the rule with least conditions) was kept. Accuracy, support and decision coverage were calculated on the complete data set for all the rules. Rules that were below the 90 % accuracy and 30 % decision coverage thresholds were discarded. In this way we extracted a single, high quality rule-based model for each of the protein for both H1N1 and H3N2 subtype data.

### Classification of sequences

In order to classify a sequence, each rule from the model was applied on it. If the conditions of the rule matched the sequence, the rule was said to fire on the sequence. Every fired rule voted for a particular classification specified by its THEN-part. The number of votes a fired rule casted was the accuracy multiplied by the support of the rule. For a sequence several rules may fire, each casting votes in favor of the class in the THEN-part. The final classification was assigned based on the majority of votes.

Consider the rules:

1. IF P70 = S THEN host = Avian. Acc = 94.0 %. Supp = 50
2. IF P14 = M and P32 = I THEN host = Avian. Acc = 93.0 %. Supp = 43
3. IF P14 = L THEN host = Human. Acc = 100 %. Supp = 285
4. IF P57 = L THEN host = Human. Acc = 100 %. Supp = 273

Now let us assume that these four rules are applied to a sequence an it turns out that Rule 2, 3 and 4 fire for this sequence. Rule 2 will cast 40 (0.93*43) votes for class Avian while rule 2 and rule 3 will cast 285 and 273 votes in favor of class Human. So, the sequence will be classified as class Human since the number of votes is 558 versus 40.

In case of no rules fired or there was a tie in the votes, the sequences were labeled as unknown.

### Performance evaluation statistics of the rule-based models

In this study the outcome *human* was considered as a positive outcome and outcome *avian* was considered as a negative one. True positives (TP) were sequences correctly classified as coming from human hosts. True negatives (TN) were sequences correctly classified as coming from avian hosts. False positives (FP) were actually avian sequences but incorrectly classified as human sequences and false negatives (FN) were actually human sequences that were incorrectly classified as avian sequences. The performance of the models for all the proteins for both H1N1 and H3N2 was assessed by the following statistics.

Sensitivity: it is also known as the true positive rate (TPR). In our case, rate at which a model correctly identifies sequences coming from a human host is the sensitivity i.e. a sequence originally from human host and classified as coming from human hosts by the model. It is calculated with the following formula:

$$ \mathit{\mathsf{S}\mathsf{ensitivity}}\kern0.5em \left(\mathit{\mathsf{S}}\mathit{\mathsf{n}}\right) = \frac{\mathit{\mathsf{T}}\mathit{\mathsf{P}}}{\left(\mathit{\mathsf{T}}\mathit{\mathsf{P}}+\mathit{\mathsf{F}}\mathit{\mathsf{N}}\right)} $$

Specificity: Also known as the true negative rate (TNR). The rate at which the model correctly identifies avian sequences is the specificity, which is calculated by:

$$ \mathit{\mathsf{S}\mathsf{pecificity}}\kern0.5em \left(\mathit{\mathsf{S}}\mathit{\mathsf{p}}\right) = \frac{\mathit{\mathsf{T}}\mathit{\mathsf{N}}}{\left(\mathit{\mathsf{F}}\mathit{\mathsf{P}}+\mathit{\mathsf{T}}\mathit{\mathsf{N}}\right)} $$

Matthews correlation coefficient: It is a measure of how well a model classifies as a whole. The difference with accuracy is that unlike accuracy Matthews correlation coefficient is not affected by un-balanced data and hence gives a better overall idea of how well the model is classifying. It is calculated by the following formula:

$$ \mathit{\mathsf{M}\mathsf{atthews}}\kern0.5em \mathit{\mathsf{correlation}}\kern0.5em \mathit{\mathsf{coefficient}}\kern0.5em \left(\mathit{\mathsf{M}}\mathit{\mathsf{C}}\mathit{\mathsf{C}}\right) = \frac{\left(\mathit{\mathsf{T}}\mathit{\mathsf{P}} \times \mathit{\mathsf{T}}\mathit{\mathsf{N}}\right)\kern0.5em -\kern0.75em \left(\mathit{\mathsf{F}}\mathit{\mathsf{P}} \times \mathit{\mathsf{F}}\mathit{\mathsf{N}}\right)}{\sqrt{\left(\mathit{\mathsf{T}}\mathit{\mathsf{P}}\kern0.5em +\kern0.5em \mathit{\mathsf{F}}\mathit{\mathsf{P}}\right)\kern0.5em \times \kern0.5em \left(\mathit{\mathsf{T}}\mathit{\mathsf{P}}\kern0.5em +\kern0.5em \mathit{\mathsf{F}}\mathit{\mathsf{N}}\right)\kern0.5em \times \kern0.5em \left(\mathit{\mathsf{T}}\mathit{\mathsf{N}}\kern0.5em +\kern0.5em \mathit{\mathsf{F}}\mathit{\mathsf{P}}\right)\kern0.5em \times \kern0.5em \left(\mathit{\mathsf{T}}\mathit{\mathsf{N}}\kern0.5em +\kern0.5em \mathit{\mathsf{F}}\mathit{\mathsf{N}}\right)}} $$

### From alignment positions to true positions

In this study the aa positions for all the H3N2 proteins except the PB1-F2 corresponds to the positions of the *A/Victoria/JY2/1968* virus. For all but PB1-F2 proteins of the H1N1 data, the positions shown in this study correspond to positions on the *A/Wisconsin/301/1976* virus. The PB1-F2 protein for both viruses is in a truncated form and we wanted to show positions from a full-length protein. For this reason we mapped the PB1-F2 H3N2 positions to the PB1-F2 of the *A/New York/674/1995* virus and the PB1-F2 H1N1 positions to full-length PB1-F2 of the *A/duck/Korea/372/2009 *virus.

### Phylogenetic analysis

FastTree 2.1.8 [44] was used to create the phylogeny trees.

### Scripting programming language

Python was used for scripting purposes.

## Abbreviations

aa, amino acids; CAG, combinatorial accuracy gain; HA, hemagglutinin; HSSs, host-specific signatures; IAVs, influenza A viruses ; LHS, left hand side; M1, matrix protein 1; M2, matrix protein 2; MCC, Matthews correlation coefficient; MCFS, Monte carlo feature selection; NA, neuraminidase; NEP, nuclear export protein; NP, nucleoprotein; NS1, non structural protein 1; NS2, non structural protein 2; PA, polymerase acidic protein; PB1, polymerase basic protein 1; PB2, polymerase basic protein 2; Sn, sensitivity; Sp, specificity

## Additional files

Additional file 1: This file contains the lists of significant features that were selected by MCFS for all the proteins of both subtypes. (XLSX 74 kb) Additional file 2: This file contains the rule-based models for all the proteins of both subtypes. (XLSX 41 kb) Additional file 3: This file contains list of names of the unseen viral sequences for both subtypes that were either miss-classified or could not be classified by the rule-based models. (XLSX 10 kb) Additional file 4: This file contains singular and combinatorial signatures from the rules for both subtypes. (XLSX 63 kb) Additional file 5: This file contains all the phylogeny trees, separate for subtype and host, marked with top 5 rules. Each sequence is represented by its GeneBank accession. The nodes with a LightBlue background are the new, unseen sequences. The unmarked nodes do not support the top 5 rules, and were either supporting rules other than the top 5 or were not classified by the models. (PDF 7180 kb) Additional file 6: This file contains all the combined subtypes and hosts phylogeny trees for each protein. Each sequence is represented by its GeneBank accession, its subtype and its host. (PDF 8113 kb)
