# Supplementary figures and images for: Identification of combinatorial host-specific signatures with a potential to affect host adaptation in influenza A H1N1 and H3N2 subtypes
Source: BMC Genomics. 2016 Jul 29;17:529. doi: 10.1186/s12864-016-2919-4 (PMC4966792; doi:10.1186/s12864-016-2919-4)

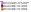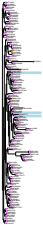

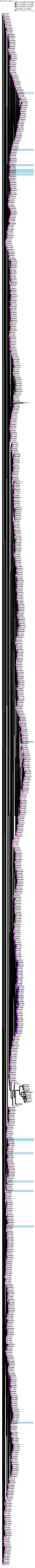

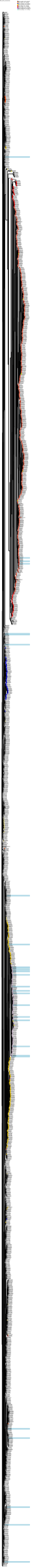

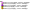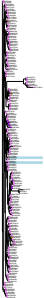

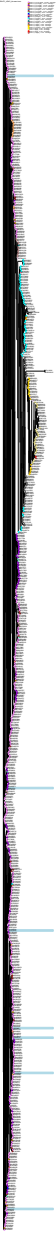

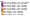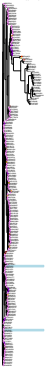

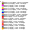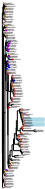

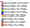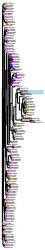

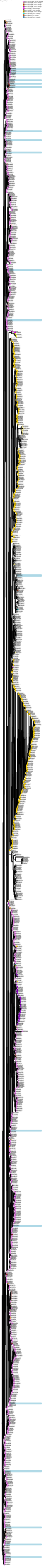

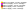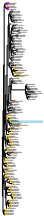

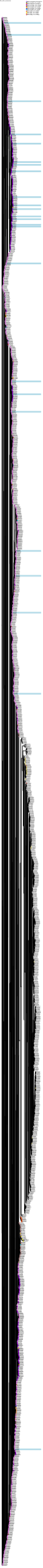

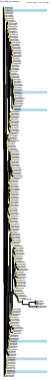

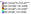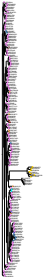

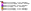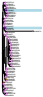

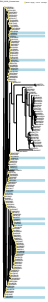

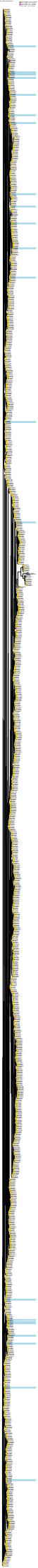

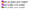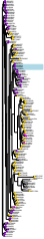

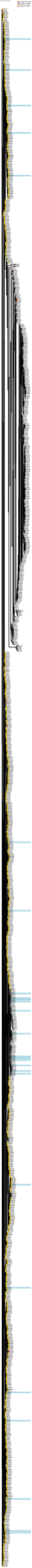

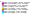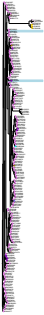

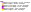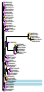

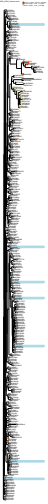

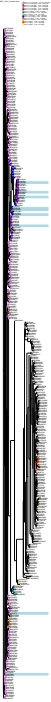

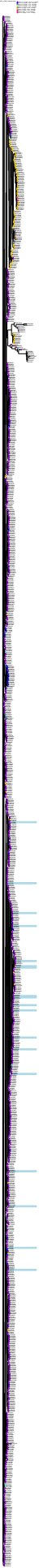

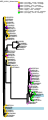

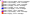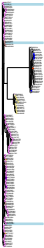

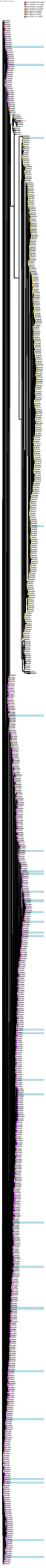

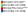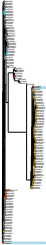

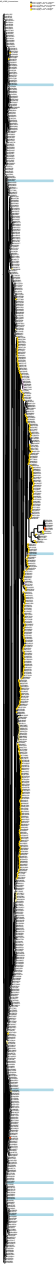

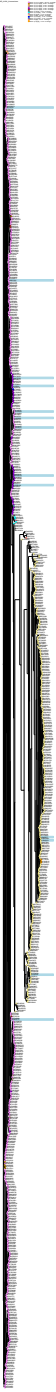

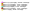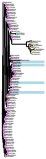

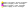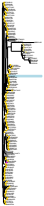

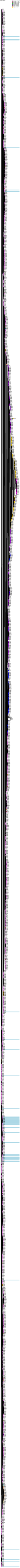

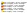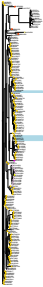

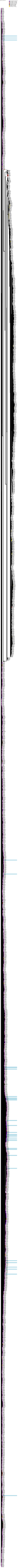

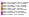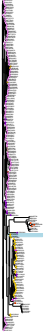

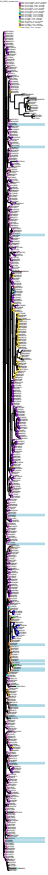

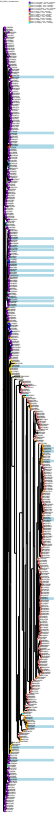

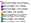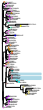

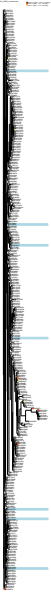

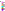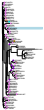

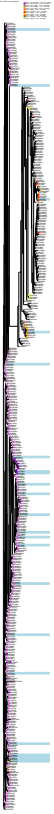

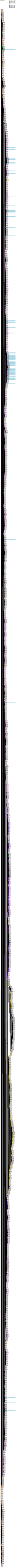

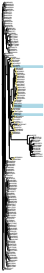

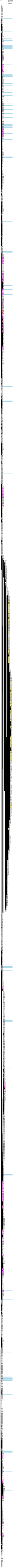

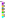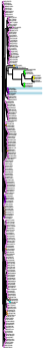

Supplement: Additional file 5: — This file contains all the phylogeny trees, separate for subtype and host, marked with top 5 rules. Each sequence is represented by its GeneBank accession. The nodes with a LightBlue background are the new, unseen sequences. The unmarked nodes do not support the top 5 rules, and were either supporting rules other than the top 5 or were not classified by the models. (PDF 7180 kb) [file 12864_2016_2919_MOESM5_ESM.pdf]

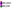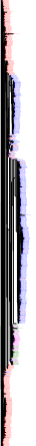

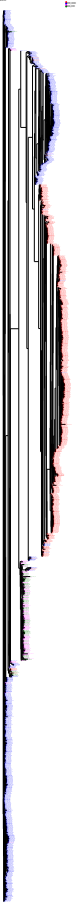

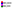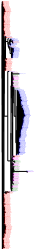

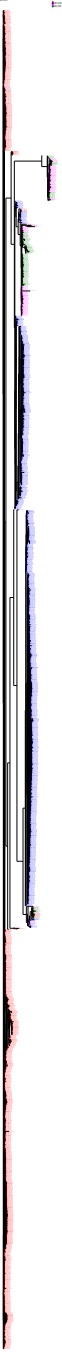

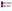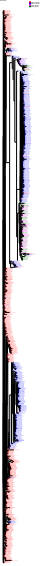

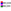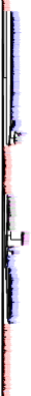

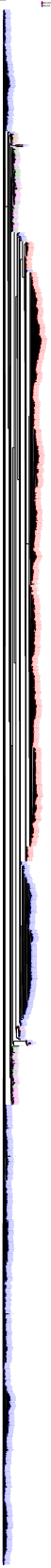

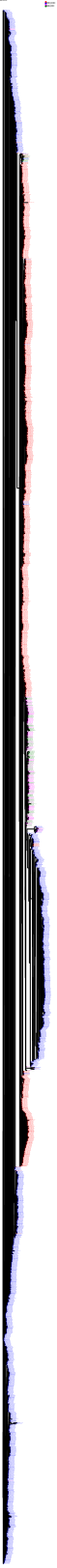

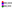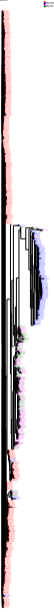

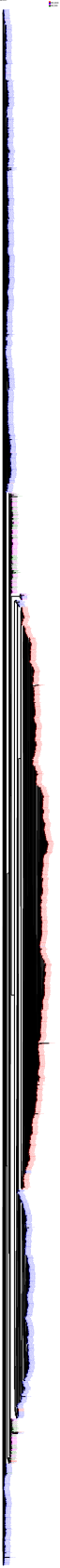

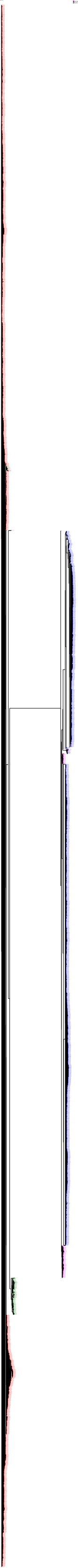

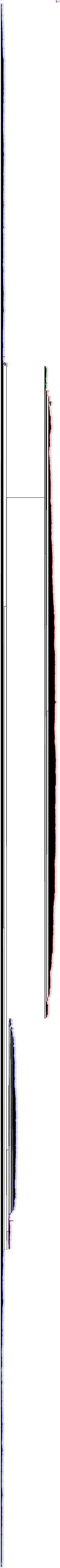

Supplement: Additional file 6: — This file contains all the combined subtypes and hosts phylogeny trees for each protein. Each sequence is represented by its GeneBank accession, its subtype and its host. (PDF 8113 kb) [file 12864_2016_2919_MOESM6_ESM.pdf]
